# Worker reproduction of the invasive yellow crazy ant *Anoplolepis gracilipes*

**DOI:** 10.1186/s12983-017-0210-4

**Published:** 2017-05-08

**Authors:** Ching-Chen Lee, Hirotaka Nakao, Shu-Ping Tseng, Hung-Wei Hsu, Gwo-Li Lin, Jia-Wei Tay, Johan Billen, Fuminori Ito, Chow-Yang Lee, Chung-Chi Lin, Chin-Cheng (Scotty) Yang

**Affiliations:** 1 Department of Biology, National Changhua University of Education, No. 1, Jin-De Rd., Changhua, 50007 Taiwan; 2 Master Program for Plant Medicine, National Taiwan University, No.1, Sec. 4, Roosevelt Rd., Taipei, Taiwan 106; 3 Faculty of Agriculture, Kagawa University, Ikenobe, Miki, 761–0795 Japan; 4 Department of Entomology, National Taiwan University, No.1, Sec. 4, Roosevelt Rd., Taipei, Taiwan 106; 5 Research Institute for Sustainable Humanosphere, Kyoto University, Gokasho, Uji, Kyoto, 611-0011 Japan; 6 Department of Entomology, University of California, Riverside, CA 92521 USA; 7 K.U. Leuven, Zoological Institute, Naamsestraat 59, box 2466, B-3000 Leuven, Belgium; 8 Urban Entomology Laboratory, Vector Control Research Unit, School of Biological Sciences, Universiti Sains Malaysia, 11800 Penang, Malaysia

**Keywords:** *Anoplolepis gracilipes*, arrhenotokous parthenogenesis, physogastric workers, trophic eggs

## Abstract

**Background:**

Reproductive division of labor is one of the key features of social insects. Queens are adapted for reproduction while workers are adapted for foraging and colony maintenance. In many species, however, workers retain functional ovaries and can lay unfertilized male eggs or trophic eggs. Here we report for the first time on the occurrence of physogastric workers and apparent worker reproduction in the invasive yellow crazy ant *Anoplolepis gracilipes* (Fr. Smith). We further examined the reproductive potential and nutritional role of physogastric workers through multidisciplinary approaches including morphological characterization, laboratory manipulation, genetic analysis and behavioral observation.

**Results:**

Egg production with two types of eggs, namely reproductive and trophic eggs, by physogastric workers was found. The reproductive egg was confirmed to be haploid and male-destined, suggesting that the workers produced males via arrhenotokous parthenogenesis as no spermatheca was discovered. Detailed observations suggested that larvae were mainly fed with trophic eggs. Along with consumption of trophic eggs by queens and other castes as part of their diet, the vital role of physogastric workers as “trophic specialist” is confirmed.

**Conclusion:**

We propose that adaptive advantages derived from worker reproduction for *A. gracilipes* may include 1) trophic eggs provisioned by physogastric workers likely assist colonies of *A. gracilipes* in overcoming unfavorable conditions such as paucity of food during critical founding stage; 2) worker-produced males are fertile and thus might offer an inclusive fitness advantage for the doomed orphaned colony.

**Electronic supplementary material:**

The online version of this article (doi:10.1186/s12983-017-0210-4) contains supplementary material, which is available to authorized users.

## Background

One of the hallmarks of higher social Hymenopterans (social bees, wasps, and ants) is the reproduction division of labor among nest members [1]. Queens are the reproductive caste that is morphologically adapted for dispersal and reproduction while workers are the non-reproductive caste specialized in foraging, nest maintenance and brood tending. A haplodiploid sex determination system is common to all hymenopterans, in which males arise parthenogenetically from unfertilized eggs (arrhenotoky) and are haploid, whereas females arise from fertilized eggs and are diploid [2, 3]. Such unique system results in an asymmetrical genetic relatedness among the colony members where workers are more genetically related to the queen’s daughters (their sisters) (*r* = 0.75) compared to their own daughters and sons (*r* = 0.50) in a monogynous colony headed by a singly mated queen [4]. According to Hamilton’s kin-selection theory, this unusual asymmetry in relatedness appears to favor evolution of a sterile worker caste as workers gain indirect fitness (i.e., propagation of their own genes) by behaving altruistically and assisting in raising the queen’s instead of their own offspring.

Reproductive constraints impair the worker reproduction either through behavioral mechanisms (e.g., worker policing) or by suppressing the development of the reproductive organs in workers [5]. However, the workers in most ant species retain functional ovaries, and are capable of producing viable male eggs and/or non-viable trophic eggs [6]. Bourke [7] reported that workers produce males in approximately 50 species from 24 genera. Trophic eggs are nutritional packets, and act as an important mechanism for transferring nutrients or protein to the colony members, especially queens and larvae (reviewed in Wheeler [8]). Nevertheless, workers that have completely lost their reproductive organs only occur in a few genera (9 out of 283). These are *Solenopsis*, *Monomorium*, *Tetramorium*, *Hypoponera*, *Anochetus*, *Leptogenys*, *Pheidole* and *Carebara* [1, 9, 10]. It is interesting to note that in primitive ant species (e.g., Ponerinae), workers possess a spermatheca, and are capable of mating and produce fertilized eggs (i.e., gamergates) [11].

The yellow crazy ant *Anoplolepis gracilipes* has been listed as one of the world’s top 100 invasive species due to their severe impacts on biological diversity and ecosystem sustainability [12]. This species is polygynous and forms supercolonies with individuals in physically separated colonies exhibiting limited aggression behavior towards each other [13]. *A. gracilipes* decimated over one-third of the entire population of endemic red crabs (*Gecarcoidea natalis*) in Christmas Island [14]. The displacement of native “keystone” species by this invasive ant indirectly impedes the litter breakdown process and causes the growth of sooty molds in canopy trees, which ultimately alters the island rainforest ecosystem. The numerical dominance of *A. gracilipes* negatively impacts the diversity and abundance of native invertebrate communities in introduced areas [15]. In addition, this species also attacks and kills populations of smaller vertebrates such as birds or new-born domestic animals, e.g. on the Seychelles [16–18].

So far, most of the well-studied invasive ants are known to possess a sterile worker caste [7], except for one previous study in which the presence of underdeveloped ovaries (i.e., absence of mature oocytes) was reported in a minority of *A. gracilipes* workers inspected [19]. While this study found little support for worker reproduction of *A. gracilipes*, our preliminary observation, in contrast, suggested that egg production often occurred in queenless *A. gracilipes* laboratory colonies, and that artificially-orphaned colonies are invariably found with the presence of “corpulent” workers, whose gaster sizes were conspicuously greater than those of “normal” foraging workers and appeared brown-whitish in color (hereafter referred to as “physogastric workers”). Such morphological difference leads to a possible link between the egg production and presence of physogastric workers, and merits further investigation. In this study, we therefore conducted a series of experiments addressing the following questions: 1) are physogastric workers present in queenright field colonies? 2) what is the anatomy of the reproductive organs of physogastric workers? 3) can *A. gracilipes* workers produce viable and/or trophic eggs under queenless condition? 4) if viable eggs are produced, what is the sex and ploidy level of such worker-produced offspring? In addition to understand the fundamental aspects of worker reproduction by *A. gracilipes*, the origin, trophic function and evolution of worker reproduction in this invasive ant species also are discussed.

## Results

### Occurrence of physogastric workers and ovarian morphology of workers

**Fig. 1:**
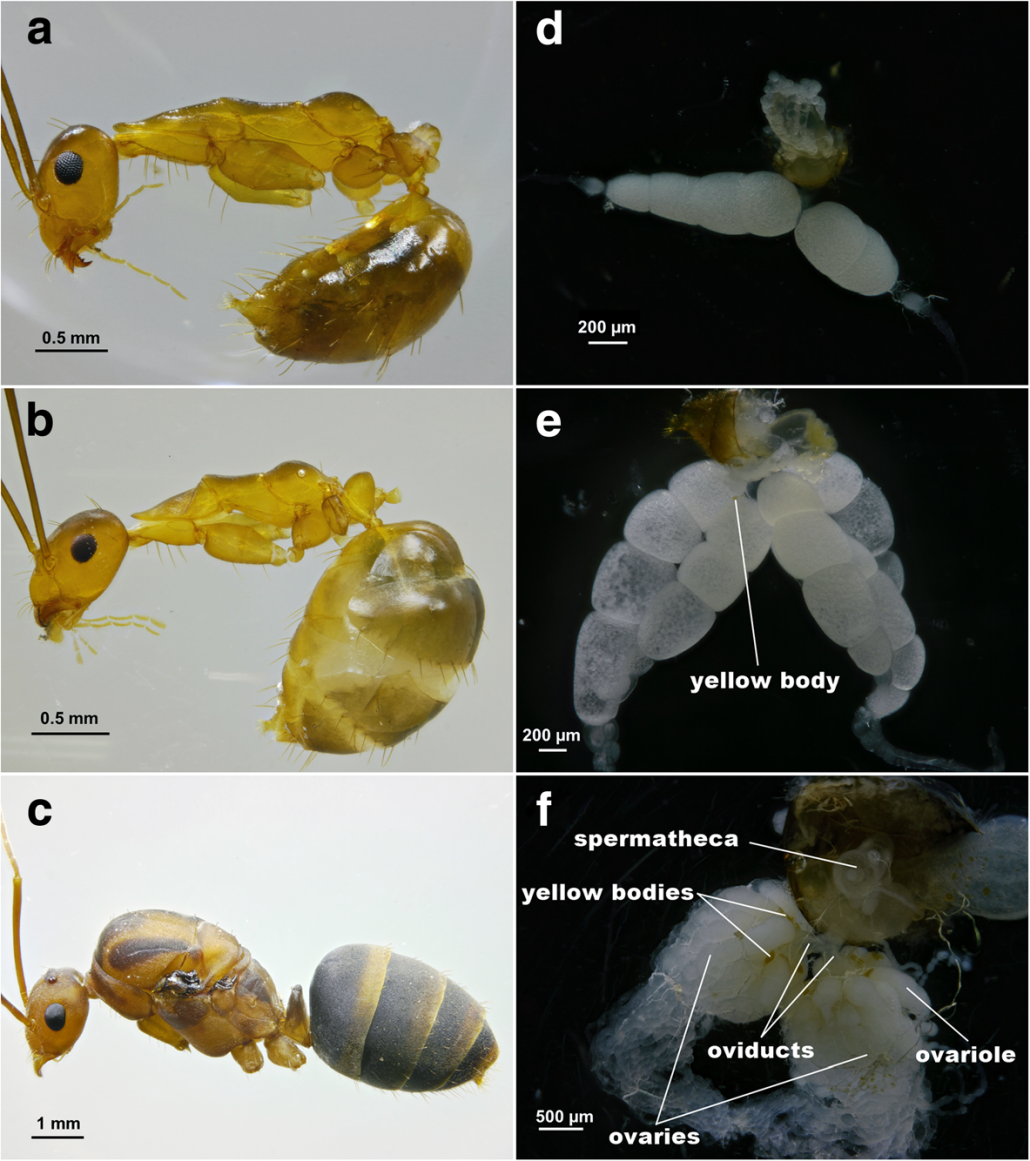
Morphology and reproductive systems in worker and queen of *A*. *gracilipes*. Shown are the external morphology of normal worker (**a**), physogastric worker (**b**) and queen (**c**). Gaster dissection presenting ovarian morphology of normal worker (**d**), physogastric worker (**e**) and queen (**f**). Note difference in length of ovarioles and number of mature oocytes

In all three field-collected colonies, 7.23–11.74% of the workers were physogastric. Gaster widths of normal workers (GW: 1.09 ± 0.03 mm, Fig. 1a) were significantly smaller than those of physogastric workers (GW: 1.53 ± 0.02 mm, Fig. 1b; Z = −5.475, *P* < 0.01; Table 1). The clearly distinct external morphology of the queen is also illustrated in Fig. 1c.

**Table 1:** Differences in gaster size and ovary development across three castes of *A. gracilipes*

Female castes	Ants dissected	Gaster width (mm)	Number of ovarioles/individual	Number of yolky oocytes per ovariole	Total number of yolky oocytes
Normal workers	*n* = 90	1.09 ± 0.03 [0.90–1.30]	1.62 ± 0.12 [1–4]	1.73 ± 0.12 [1–4]	2.81 ± 0.31 [1–8]
Physogastric workers	*n* = 90	1.53 ± 0.02 [1.40–1.70]	2.51 ± 0.09 [2–5]	4.46 ± 0.10 [1–9]	11.21 ± 0.48 [6–27]
Z		-5.475	−5.652	−10.416	-8.290
*P*		* < 0.01	* < 0.01	* < 0.01	* < 0.01
Queens	*n* = 9	2.77 ± 0.06 [2.60–3.00]	47.33 ± 1.23 [44–52]	2.00 ± 0.04 [1–3]	94.50 ± 6.63 [69–116]

Notes: Data are presented as mean ± standard deviation [range]; *p, statistically significant using Mann-Whitney *U*-test; Queens were not subjected to analysis due to its small sample size

We found normal workers possess ovaries, most of which, however, are underdeveloped and lacking of yolky oocytes (92%) (Fig. 1d). Physogastric workers tend to possess more well-developed ovaries (Fig. 1e) as the number of ovarioles/individual is higher than in normal workers (2.51 ± 0.09 vs. 1.62 ± 0.12; Z = −5.652, *P* < 0.01; Table 1), the number of yolky oocytes per ovariole (4.46 ± 0.10 vs. 1.73 ± 0.12; Z = −10.416, *P* < 0.01) and the total number of yolky oocytes were significantly higher in physogastric workers than in normal workers (11.21 ± 0.48 vs. 2.81 ± 0.31; Z = −8.290, *P* < 0.01). Note that numbers presented here were based on those ovarioles with at least a visible oocyte only. While no spermatheca was found in both types of the workers, yellow bodies that are characteristic of reproduction were visible in some physogastric workers (13%) (Fig. 1e). On average, queens of *A. gracilipes* had 44–52 ovarioles/individual and had a higher number of yolky oocytes (94.50 ± 6.63) than both types of workers. Yellow bodies were present in the ovaries of queens, along with a conspicuous spermatheca (Fig. 1f).

### External and internal morphology of workers

**Fig. 2:**
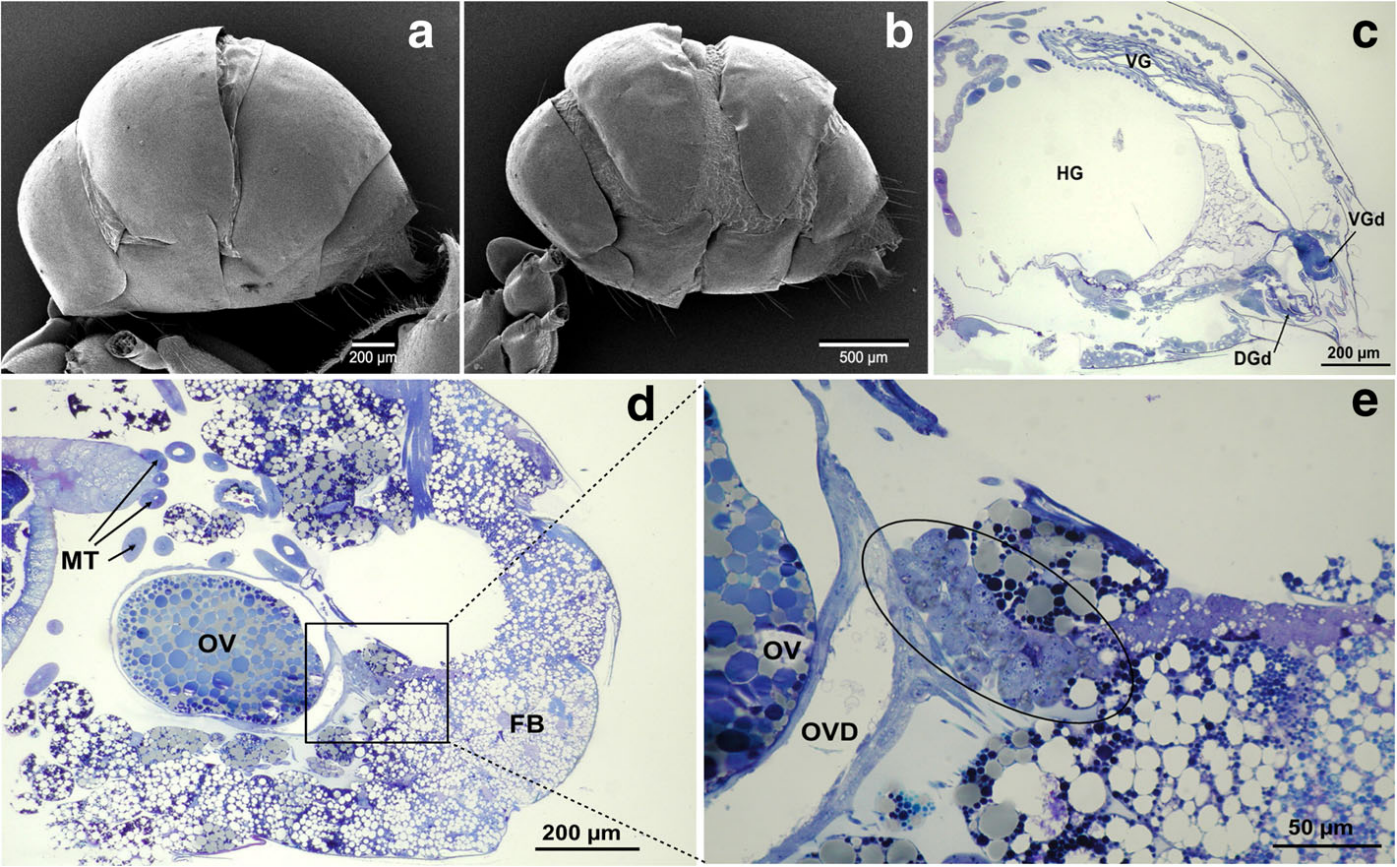
Scanning electron micrographs and histological sections of two types of workers in *A*. *gracilipes*. SEMs of abdomen of normal worker (**a**) and physogastric worker (**b**), and longitudinal sections through posterior abdomen part of normal worker (**c**) and physogastric worker (**d**). Note large accumulation of fat body and absence of spermatheca in physogastric worker. The location where spermatheca is supposed to be found if it exists is highlighted with circled area in figure (**e**). DGd: Dufour gland duct, FB: fat body, HG: hindgut, MT: Malpighian tubules, OV: ovaries, OVD: oviduct, VG: venom gland, VGd: venom gland duct

Scanning electron microscopy revealed a noticeable difference in abdominal morphology between normal and physogastric workers (Fig. 2a and b). The abdomen of physogastric workers was greatly distended with exposed intersegmental membranes. Histological sections indicated that the fat body in the abdomen is far more abundant in physogastric than in normal workers (Fig. 2c and d). The absence of a spermatheca in physogastric workers was further confirmed by longitudinal histological sections (Fig. 2e), suggesting that sexual reproduction by workers of *A. gracilipes* is impossible.

### Production of eggs by workers, sex, ploidy level and morphology of worker-produced offspring

**Fig. 3:**
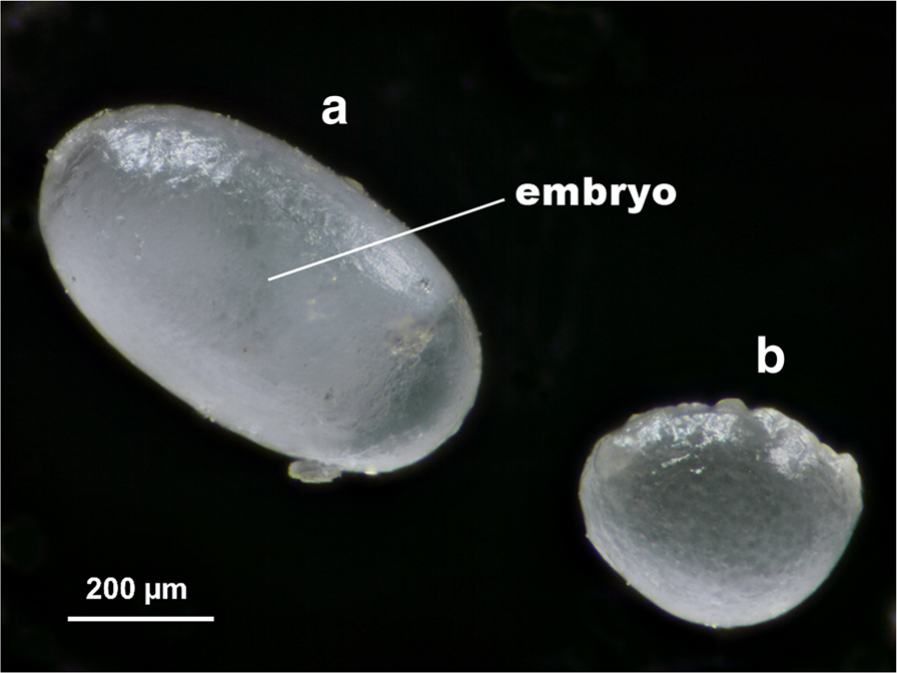
Morphology of eggs produced by *A*. *gracilipes* workers. Light micrograph of a worker-laid reproductive egg (**a**) and a worker-laid trophic egg (**b**)


After 4 months, we discovered that three out of nine artificially-orphaned colony fragments produced eggs and larvae, these three colonies fragments were designated as AGQLF01, AGQLF02 and AGQLF03. AGQLF01 was isolated from the colony in Nantou County, while both AGQLF02 and AGQLF03 were isolated from the same source colony in Changhua County (Additional file 1: Figure S1). Morphological observations indicated the existence of two types of eggs produced by the workers, characteristically elongated oval shaped eggs and sub-spherically shaped eggs (Fig. 3). The former was confirmed viable with an obvious embryo, whereas the latter was embryoless and never hatched. Coupled with the fact that these non-viable eggs are consumed by larvae (Additional file 2: Video S1) and other castes (see “Fate of worker-laid trophic eggs” for more details), it is most likely that the eggs with sub-spherical shape serve as trophic eggs. Reproductive eggs in one (AGQLF03) of the three egg-producing colonies successfully developed into pupae that emerged as adult males (*n* = 18) 6 months after the start of the experiment, confirming the viability of the elongated oval shaped eggs. While larvae were present in AGQLF01 and AGQLF02 during the first 4 months, we failed to recover any adult male upon the end of the observation most likely due to cannibalism by nestmate (see Discussion).

Additional file 2: **Video S1.** Fate of trophic eggs. A physogastric worker (2^nd^ worker in the upper left-hand corner) bends its gaster forward, seizes the freshly-laid egg with mandible and immediately offers the egg to an adjacent larvae pile. The video can be accessed through the URL https://www.youtube.com/watch?v=SvyrSZ-4n-s&feature=youtu.be. (MOV 2973 kb)


Results of microsatellite genotyping revealed that all workers from AGQLF03 are heterozygotes across all loci with the presence of three major representing multi-locus genotypes (Table 2). Unlike the previously reported high frequency of heterozygous males [19, 20], we found that all males from AGQLF03 possess homozygous multi-locus genotypes, harboring one of the maternal alleles at all loci, which suggests that the worker-produced males are invariably haploid. In contrast, virtually all males (90%) in the queenright colony (AGQR01) are diploid (heterozygous at least at one locus), a pattern consistent to previous studies that diploid males are common in the introduced ranges [19, 20].

**Table 2:** Genotypic distribution for individuals of various castes (queen, workers and males) from a queenright and queenless colony

Caste	Sample size	*Ano1*		*Ano3*		*Ano4*		*Ano5*		*Ano6*		*Ano8*		*Ano10*	
*AGQR01* (queenright colony)															
Queen	1	118		168		171		139		133		232		306	
Worker	1	112	118	152	168	173	177	121	139	133	145	210	232	262	306
Worker	2	112	118	152	178	171	177	121	135	133	145	210	232	262	306
Worker	2	112	118	152	168	171	177	121	135	133	145	210	232	262	290
Worker	3	112	118	152	168	171	177	121	135	133	145	212	234	262	308
Worker	1	112	118	152	168	171	177	121	135	133	145	212	234	262	292
Worker	3	112	118	152	178	173	177	121	135	133	145	210	232	262	306
Worker	1	112	118	152	178	173	177	121	139	133	145	210	232	262	306
Worker	1	112	118	152	178	173	177	121	135	133	145	212	234	262	308
Worker	1	112	118	152	168	173	177	121	135	133	145	212	234	262	308
Male	2	112	118	152	178	171	177	121	135	133	145	210	232	262	306
Male	1	112	118	152	168	171	177	121	135	133	145	210	232	*−1*	*−1*
Male	1	112	118	152	178	173	177	121	135	133	145	210	232	262	290
Male	1	112	118	152	168	171	177	121	135	133	145	210	232	262	306
Male	2	112	118	152	168	171	177	121	135	133	145	212	234	262	308
Male	1	112	118	152	178	173	177	121	135	133	145	212	234	262	308
Male	1	112	118	152	168	173	177	121	135	133	145	212	234	262	308
Male	1	112	118	152	178	173	173	121	135	133	145	210	232	262	306
Male	1	112	118	152	178	171	177	121	135	133	145	212	234	*−1*	*−1*
Male	1	112	118	152	178	171	177	121	135	*−1*	*−1*	212	234	262	308
Male	1	112	118	152	168	171	177	121	135	*−1*	*−1*	212	234	262	308
Male	1	112	118	152	178	177	177	121	135	133	145	210	232	262	262
Male	1	112	118	152	168	177	177	121	135	133	145	210	232	*−1*	*−1*
Male	1	112	118	152	178	177	177	121	135	133	145	212	234	262	308
Male	1	112	118	152	168	177	177	121	135	133	145	212	240	262	292
Male	1	112	118	152	168	177	177	*−1*	*−1*	145	145	210	210	*−1*	*−1*
Male	1	112		152		177		121		145		*−1*		262	
Male	1	112		152		177		121		145		210		*-1*	
*AGQLF03* (queenless colony fragment)															
Worker	4	112	118	152	168	171	177	121	139	133	145	212	212	262	290
Worker	9	112	118	152	168	171	177	121	139	133	145	210	210	262	288
Worker	1	112	118	152	168	171	177	121	139	133	145	210	210	262	290
Male	1	118		152		171		139		133		*−1*		290	
Male	2	112		168		171		121		145		212		290	
Male	1	118		152		177		139		145		*−1*		292	
Male	1	118		168		171		121		145		*−1*		262	
Male	1	118		168		171		139		145		210		288	
Male	1	112		168		177		139		145		210		262	
Male	1	112		168		177		121		145		210		290	
Male	1	118		168		171		121		133		*−1*		262	
Male	1	118		152		177		139		133		*−1*		288	
Male	1	112		168		177		139		145		*−1*		288	
Male	1	112		152		171		121		145		*−1*		290	
Male	1	118		168		177		121		133		*−1*		262	
Male	1	112		168		171		121		133		210		262	

*−1*, amplification failure

**Fig. 4:**
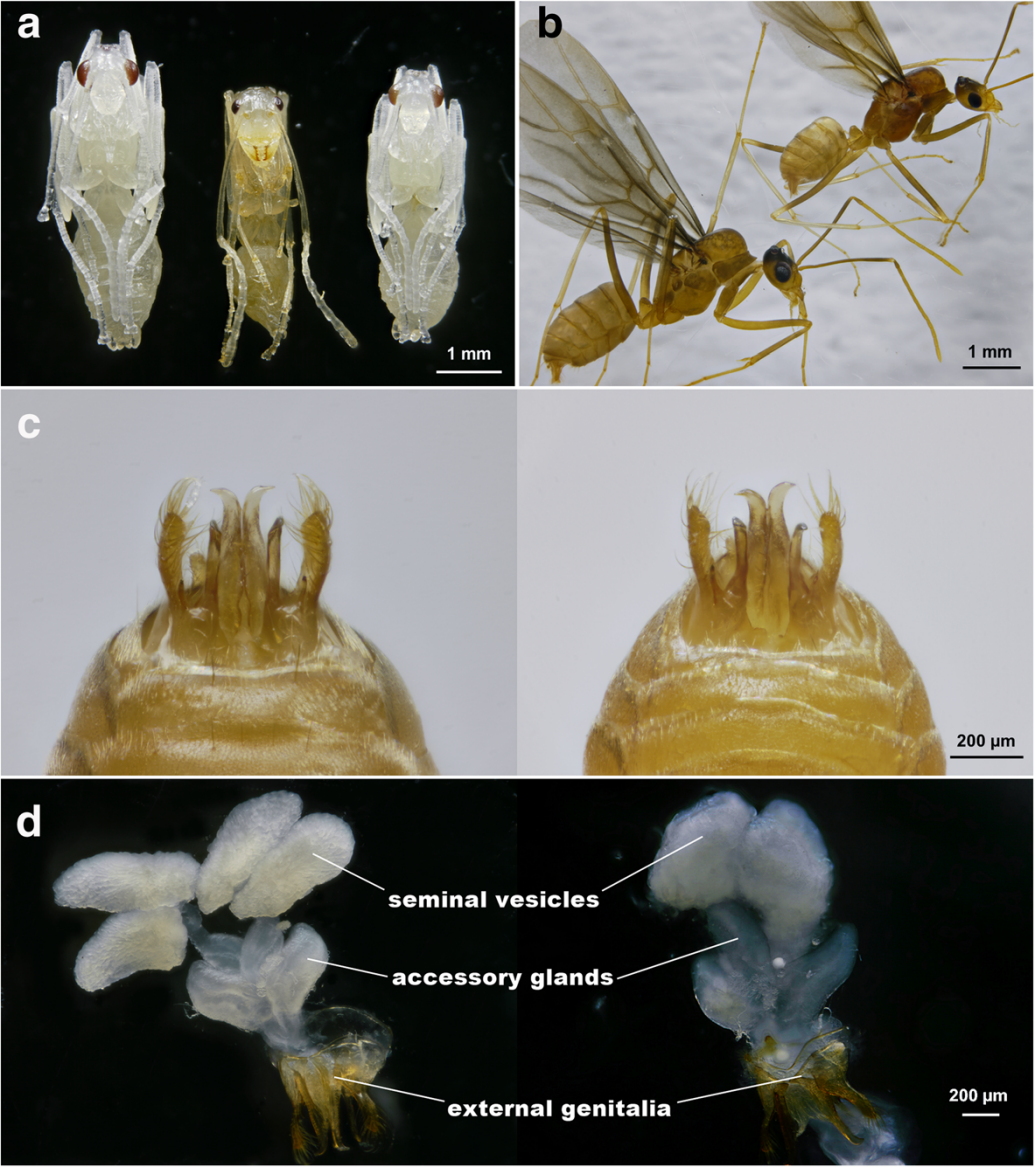
Morphological comparison between worker-produced and queen-produced offspring in *A*. *gracilipes*. **a** Male pupa from orphaned colony (*left*), worker pupa (*middle*) and male pupa from queenright colony (*right*); (**b**) external morphology of worker-produced male (*left*) and normal queen-produced male (*right*); (**c**) close up of external genital structure of worker-produced male (*left*) and queen-produced male (*right)*; (**d**) internal organs of the male reproductive system of worker-produced male (*left*) and queen-produced male (*right*)


Both head width and total body length of worker-produced male pupae (HW: 0.81 ± 0.01 mm; TL: 4.06 ± 0.07 mm) were significantly greater than those of queen-produced male pupae (HW: 0.70 ± 0.01 mm; TL: 3.69 ± 0.05 mm; Fig. 4a; HW: Z = −3.888, *P* < 0.01; TL: Z = −3.060, *P* < 0.01). Similarly, the two measurements of worker-produced males (HW: 0.80 ± 0.02 mm; TL: 4.58 ± 0.10 mm) were also greater than those of queen-produced males, respectively (HW: 0.71 ± 0.01 mm; TL: 4.00 ± 0.05 mm; Fig. 4b; HW: Z = −2.985, *P* < 0.01; TL: Z = −2.863, *P* < 0.01). Worker-produced males, however, shared similar genital structures (Fig. 4c) and had similar internal reproductive organs (Fig. 4d) as adult males in a queenright colony. Rupturing of seminal vesicles in worker-produced males further showed the presence of viable sperm (i.e., sperm bundle with apparent swimming ability, Additional file 3: Video S2).

Additional file 3: **Video S2.** Sperm bundles. Motile sperm bundles in the seminal vesicle of worker-produced males. The video can be accessed through the URL https://www.youtube.com/watch?v=-AfHtSnak6A. (MOV 4352 kb)


### Fate of worker-laid trophic eggs

We tracked the fate of 62 and 51 trophic eggs produced by physogastric workers in colonies AGTE01 and AGTE02, respectively (Table 3). Visual observations indicated that most of the trophic eggs (≥ 63%) were offered to the larvae. Coupled with the fact that larvae received occasional trophallaxis from workers and never directly fed on solid prey items during the entire observation period, trophic eggs appear to be the main food source for larvae in *A. gracilipes*. Queens received both liquid food via oral trophallaxis and trophic eggs from workers, and the former seems to be their main diet (≥ 89%; Table 4). We also discovered that trophic eggs were occasionally offered to other castes such as workers and males.

**Table 3:** The fate of worker-laid trophic eggs expressed by which caste/stage was a given trophic egg offered to after being laid

	Colony code	
	AGTE01	AGTE02
Number of queens	1	2
Number of workers	≈ 900	≈ 250
Total hours of observation	10.5	10
Total number of trophic eggs that had been followed	62	51
Number of trophic eggs given to		
dealate queens	3 (5%)	4 (8%)
males	-	8 (15%)
workers	3 (5%)	1 (2%)
larvae	56 (90%)	25 (49%)
queen larvae	-	7 (14%)
Number of trophic eggs not given to any of particular caste mentioned above	-	6 (12%)

Notes: Values in parentheses refer to proportion of trophic eggs given to respective individuals or castes relative to the total number of trophic eggs that we tracked; Neither males nor queen larvae were found in colony AGTE01 during the observation

**Table 4:** Dietary composition (trophallaxis vs. consumption of trophic egg) of queens of *A. gracilipes*

	Colony code & individual queen		
	AGTE01 Q1	AGTE02 Q1	AGTE02 Q2
Total hours of observation	10.5	10.5	10.5
Feeding on			
liquid food via oral trophallaxis from workers	324 times (89%)	74 times (97%)	60 times (92%)
trophic eggs	40 times (11%)	2 times (3%)	5 times (8%)

Notes: Values in parentheses refer to frequency of consumption relative to the total number of feedings by queens; one whole trophic egg was consumed per feeding

## Discussion

We performed both field survey and laboratory manipulation to study worker reproduction in the invasive yellow crazy ant, *A. gracilipes*. The results of our survey confirm the existence of physogastric workers in the field colonies, and subsequent gaster dissection reveals that the level of ovarian development is significantly higher in physogastric than in normal workers. Workers in artificially orphaned colonies produced both trophic and viable (reproductive) eggs. The viable eggs from one of the queenless colonies successfully developed into males that were slightly larger than those produced by queens. All worker-produced males were haploid and possess a normal, functional reproductive system as their diploid counterparts do. Furthermore, our data suggest that the production of trophic eggs plays a crucial role in regulating colony nutrition, especially for larvae. Below we discuss how these findings, combined with additional evidence obtained from histological, SEM and behavioral observation, provide new insights into the role of physogastric workers in *A. gracilipes*.

### Arrhenotokous parthenogenesis by physogastric workers

Our results clearly demonstrate that *A. gracilipes* workers are not functionally sterile, yet able to produce both trophic and reproductive eggs. In several ant species, workers are known for their ability to produce trophic eggs in queenright colonies and switch to produce reproductive eggs which develop into males once the queens die or disappear [21–23]. For instance, *Aphaenogaster senilis* workers produce unviable trophic eggs under queenright condition and begin to lay reproductive eggs that develop into males 4 months after being separated from the queens [24]. Similar to previous studies, our work showed that approximately 6 months after queen removal, some viable reproductive eggs successfully developed into adult males in one of the experimental *A. gracilipes* colony fragments. To the best of our knowledge, this is the first study showing that worker reproduction occurs in *A. gracilipes*.

While we lack direct evidence on whether worker-produced males can copulate with queens or female alates, their seemingly functional genitalia, intact reproductive organs and presence of viable sperm lead us to speculate that worker-produced males may have equal reproductive capacities as queen-produced males. It is thus plausible that in field conditions the last cohort of worker-produced males might be able to copulate with female alates from other colonies in the proximity, and subsequently offer fitness advantage to the doomed orphaned colony.

Previous studies have suggested that thelytokous parthenogenesis (i.e., diploid daughter females are produced from unfertilized eggs) may have occurred in *A. gracilipes* based on the finding of high intracolonial relatedness among workers [20, 25, 26]. In contrast, our microsatellite analyses suggested that worker-produced reproductive eggs are invariably haploid, instead of diploid as expected when thelytokous parthenogenesis operates. We also showed that virtually all males in the queenright colony are diploid, which is consistent with previous studies in which a high prevalence of diploid males in *A. gracilipes* colonies was discovered [19, 20]. The high proportion of diploid males in the field colonies collected from this study and elsewhere may imply that in field conditions the haploid males are either rarely produced by workers when colonies remain queenright or are mostly consumed by nestmates as food resources. In general, mechanisms (e.g., queen repression, worker self-restraint and/or worker policing [27–30]) contributing to low or absence of worker reproduction in a queenright colony are predictable with sex ratio optimization, relatedness asymmetry and/or kin structure [31]. For example, Chapuisat et al. [32] found that male larvae of *Formica exsecta* are preferentially cannibalized by nestmate workers at their late developmental stage not only to regulate the sex ratio of colony but also to feed the females as additional food. We, however, note that the presence of an unusual reproduction mode (e.g., asexual production of the queen) and a high frequency of diploid males of *A. gracilipes* [19, 20] may not satisfy the prerequisites of such prediction (e.g., classic haplodiploidy), thus leading the interpretation of a favorable scenario extremely difficult. Yellow bodies were found in some of the physogastric workers from queenright colonies in this study, however, it remains questionable whether yellow bodies can be an appropriate indicator for oviposition of viable eggs as they are also visible in trophic egg layers for some species [33–35].

Furthermore, the body size of haploid males produced by workers is, on average, greater than that of the diploid counterparts produced by queens, and such finding is opposite to what has been reported for other ant species (e.g., *Atta sexdens*, *Lasius sakagamii*, *Solenopsis invicta*) whose diploid males tend to be bigger than haploid ones due to diploidization and feminization [36–38]. Such inconsistency, however, might be explained by factors other than ploidy. Larger size and functional aspermy seem to be common feminized characteristics in diploid males of numerous hymenopterans [39]. The reproductive tracts in all diploid males of *A. gracilipes* we dissected, however, are fully functional with the presence of viable sperm, suggesting a negligible effect of ploidy level. We therefore regard the excess of food supply (to ensure worker survival since orphaned [40]) to the orphaned colony fragments or other factors such as social environment as an alternative contributing factor for the larger size of haploid males.

### Physogastric workers as trophic specialist

Our observations have suggested that trophic eggs constitute a major dietary regime for larvae and approximately 11% of dietary regime in queens, suggesting physogastric workers that account for production of trophic eggs function as a trophic specialist in *A. gracilipes* colonies. At least three additional lines of evidence support such a nutritional role of the physogastric workers. First, while consumption of trophic eggs as main diet has been widely reported in ant species lacking the ability to share resources via trophallaxis [41], trophic eggs may hold as equal nutritional value in other trophallaxis-performing ant species [40, 42]. One plausible reason among is that trophic eggs serve as an essential food source for a specific caste and/or developmental stage in the colony [8, 43]. Our data are in perfect agreement with such prediction as larvae of *A. gracilipes* appear to mainly consume trophic eggs during our entire observation period. Moreover, trophic eggs also are occasionally fed to queens, males and nestmate workers of *A. gracilipes* despite the presence of trophallaxis, further confirming that trophic eggs may serve as additional nutritional sources under some circumstances. Secondly, physogastric workers were found to occur together with younger brood and queens in the royal chamber (Additional file 2: Video S1, Additional file 4: Figure S2), and never engaged in foraging or other tasks outside the royal chamber. One may expect that trophic eggs, once produced, could be fed to the queen and larvae right away as they all stay within close proximity. This interpretation is further supported by our video showing that a trophic egg was fed to the adjacent brood pile immediately after it was laid by a physogastric worker (Additional file 2: Video S1).

The third line of evidence linked to the trophic function of physogastric workers is that the proportion of physogastric workers in the colony appears to be higher during fall and winter based on a preliminary field observation (CCLee et al., unpublished data). *A. gracilipes* is well-known for its broad diet as they prey on a variety of invertebrates as protein-rich food source (e.g., insects, small isopods and arachnids [16]). However, prey items of such kind cannot be stored easily as they are perishable, and its availability also fluctuates on a seasonal basis [43]. The high proportion of physogastric workers in the colony likely results in an increasing production of trophic eggs and thus represents an innate response of the colony to the declining availability of arthropod prey during such seasons [44]. We therefore suggest that the production of trophic eggs can be further regarded as an adaptive strategy for *A. gracilipes*, allowing colonies to sustain during unfavorable climatic conditions or periods of food shortage (e.g., winter) as trophic eggs can be stored for a longer period [45] and easily re-distributed within the colony when needed.

### Evolution of worker production in *A. gracilipes*

The presence of males in only one out of nine well-fed artificially-orphaned fragments suggests that male production by *A. gracilipes* workers after dequeening under field conditions appear to be uncommon. Such pattern might be explained by three mutually non-exclusive mechanisms: 1) If worker-laid trophic eggs are essential in terms of nutrient provision in the colony of *A. gracilipes*, selection may favor increased reproductive potential of worker castes (i.e., physogastric workers). Thus, the occasional emergence of worker-produced males could simply represent a by-product of the reproductive workers possessing highly-developed ovaries [40, 41]. 2) Theoretically, each focal reproductive worker is expected to be more closely related to her own son than to the average worker-produced sons (nephews) [4]. Under this condition, kin selection theory predicts that potential conflict will arise among physogastric workers over male parentage as all physogastric workers are able to lay male eggs and will selectively remove work-laid brood (i.e., worker policing) to which they are less related [30, 46]. Nevertheless, extraordinarily high intracolony relatedness despite polygyny nature of *A. gracilipes* and unusual reproductive system [19, 20] indicate that worker-policing or competition for male parentage, if any, in this species possibly could not be explained by relatedness alone. 3) Aside from the relatedness hypothesis, low frequency of male production by workers in *A. gracilipes* may be attributed to selection for higher worker efficiency and colony-level productivity. An increasing number of studies have proposed that the cost of worker reproduction appears to underlie the regulation of worker policing and self-restraint in social insects [47, 48]. For instance, workers showed aggression behavior toward reproductive workers in the asexually reproducing ant, *Platythyrea punctata* where genetic conflicts are not expected as colony members are identical to each other due to clonality [49]. This is because reproductive workers invest less in non-reproductive tasks and hence may reduce the entire colony efficiency by disrupting the foraging activity or reducing life span in workers [27, 50, 51]. Similarly, it is highly possible that the male brood derived from workers in *A. gracilipes* is prevented from development by worker policing for optimizing priority task of physogastric workers, that is, the provisioning of nestmates with trophic eggs or other nursery-related tasks. Our data partially support this interpretation that male brood were found in all three viable egg producing colony fragments, but only one of which was observed with the presence of adult males.

## Conclusion

Our study demonstrates for the first time that *A. gracilipes* workers possess functional ovaries and are able to produce both reproductive and trophic eggs. The former can be further developed into haploid males that may have equal reproductive fitness as their diploid counterparts, whereas the latter may have served as a critical regulator for protein-rich food (especially for larvae), thus allowing the colonies of *A. gracilipes* to survive through periods of food shortage. Furthermore, the current study offers an excellent chance to study if production of trophic eggs functions as an adaptive strategy for *A. gracilipes* when encountering food shortage, and how such behavior contributes to the success and ecological dominance of this ant as invasive species. We are currently generating the necessary baseline data to elucidate the ecological role of physogastric workers, factors that trigger ovary development of workers, the reproductive value of worker-produced males and how the combination of these mechanisms contributes to the invasiveness of this ant species.

## Methods

### Existence of physogastric workers under natural conditions and reproductive organs of *A. gracilipes* workers

Between December 2015 and February 2016, three queenright colonies of *A. gracilipe*s were collected from Nantou (AGQR01), Changhua (AGQR02) and Miaoli (AGQR03) counties, Taiwan (Additional file 1: Figure S1), and brought to the lab for further inspection and experimental manipulations. Firstly, the presence of physogastric workers was visually inspected, and the percentage of physogastric workers in each colony was assessed. We define physogastric workers as workers whose gaster size is distinctly greater than that of normal foraging workers, and that appear brown-whitish in color. Thirty physogastric and thirty normal workers were randomly selected from each colony and dissected shortly after collection in the field (between 1 and 2 weeks). Prior to dissection, we measured gaster width (GW), maximum transverse distance across the gaster in dorsal view. Workers were anaesthetized with carbon dioxide followed by pulling of the last gastral tergite by forceps in PBS solution. Fat and tissue were removed to ease subsequent observation. To determine the ovarian development of workers (both physogastric and normal ones), the number of ovarioles/individual, number of mature or yolky oocytes per ovariole, and total number of yolky oocytes were counted for each worker inspected. As immature ovarioles are threadlike and difficult to visualize during dissection, we only focused on those ovarioles with at least one visible oocyte. The presence of yellow bodies and a spermatheca was also visually inspected in both types of workers. In addition, three queens per colony (a total of nine) were dissected to characterize the anatomical differences between queen and worker.

### SEM analysis and histology

Physogastric and normal workers for scanning microscopy were critical point dried in a Balzers CPD 030 instrument, mounted on SEM-stubs, coated with gold, and examined in a JEOL JSM-6360 scanning microscope. To further confirm the presence of a spermatheca in both worker types, five physogastric and five normal workers were randomly selected from each colony (30 in total) mentioned above for histological sections. The posterior part of the gaster was cut off using microscissors and was fixed in cold 2% glutaraldehyde in a 50 mM Na-cacodylate buffer at pH 7.3 with 150 mM saccharose. After postfixation in 2% osmium tetroxide in the same buffer and dehydration in a graded acetone series, tissues were embedded in Araldite. Serial longitudinal sections with a thickness of 2 μm were made with a Leica EM UC6 ultramicrotome, stained with methylene blue and thionin and viewed in an Olympus BX-51 microscope. Voucher specimens were deposited in the Research Institute for Sustainable Humanosphere, Kyoto University, and are available upon request.

### Production of eggs in artificially-orphaned colonies

A total of three colony fragments constituted of 100 randomly-selected normal workers were separated from each of the three original nests (*n* = 9). Caution was taken to avoid transfer of eggs and brood from the original colonies to ensure the presence of eggs in the colony fragment after isolation is the result of worker reproduction. Each colony fragment was cultured in a polyethylene container (39 × 31 × 10 cm) with its edges and inner surfaces coated with a thin layer of fluon to prevent escape of ants. Sugar water (10%), crickets, and honeybee larvae were provided *ad libitum*. The experimental colony fragments were maintained under constant environmental conditions of 26 ± 1 °C, 60 ± 5% relative humidity and a 12-h photoperiod. The egg and brood production were monitored (4 months) on a weekly basis, starting 4 weeks after colony fragmentation. If eggs were found, the morphology of the egg and eventual presence of an embryo were examined under a microscope. Some of the eggs were left uncollected and allowed to develop into pupa and adult stage if possible.

### Sex, ploidy level and morphology of worker-produced offspring

If any worker-produced offspring was found at the end of the experimental period, both worker-produced offspring and several nestmate workers (randomly selected from the same colony fragment) were subjected to microsatellite genotyping. We genotyped a total of 14 worker-produced males and nestmate workers each from a queenless colony fragment﻿ (AGQLF03). To compare the genotypic distribution of individuals between queenright and queenless colony fragments, individuals of different castes including queen, workers and males from a queenright colony (AGQR01) were also genotyped. We genotyped a total of 15 workers and 20 males each from the queenright colony. Genomic DNA was extracted from tissue of each individual ant using the Gentra Puregene cell and tissue kit (Qiagen, USA) according to the manufacturer’s instructions. Individual genotypes were assessed at seven nuclear microsatellite loci, including *Ano1*, *Ano3*, *Ano4*, *Ano5*, *Ano6*, *Ano8* and *Ano10*, previously developed by Feldhaar et al. 2006 [52]. Microsatellite loci were amplified using the multiplex PCR method described by Blacket et al. 2012 [53]. The seven loci were amplified in two separate 15 μL multiplex-PCRs, each containing three to four pairs of primers (0.2 μM), 0.2 unit of SuperTherm Hot-start Taq DNA Polymerase (JMR Holdings, UK), 0.25 mM of each dNTP, 1X Super-Therm Gold PCR buffer (JMR Holdings, UK), and 10–20 ng of template DNA. Thermal cycling profiles were as follows: one cycle of 95 °C (10 min), followed by 35 cycles of 94 °C (30 s), primer-specific annealing temperature 55 °C (30s), and 72 °C (30 s), followed by a single final extension of 72 °C (30 min). The resulting PCR products were analysed on an ABI-3730 Genetic Analyzer (Applied Biosystems) by Genomics BioSci & Tech Co., Ltd. (Taipei, Taiwan). GeneMarker program (version 2.4.0, Softgenetics LLC) was employed to visualize and score alleles. Samples harboring homozygous multi-locus genotypes were considered haploid individuals, while those with heterozygote at one or more loci were considered diploid.

If any pupa or adult male successfully emerged in a worker-only colony fragment, both life stages were subjected to morphometric measurement. Pupa and male sizes were measured as head width (HW), maximum width of the head between the compound eyes and total body length (TL), and the total outstretched length from the mandibular apex to the gastral apex. The above-mentioned measuring procedures were repeated on the individuals collected from queenright colony as reference.

### Fate of worker-laid trophic eggs

A colony fragment composed by individuals from different castes was separated from each of the two original nests (AGQR01 & AGQR02; *n* = 2). Each colony fragment was maintained in a polypropylene container in which several transparent plastic boxes were inversely placed for housing ants as nest chambers [54]. The bottom of the container was filled with moistened plaster of Paris. These two colony fragments were designated as AGTE01 and AGTE02. A nest chamber was randomly selected for observation. Egg-laying workers or workers carrying trophic eggs were identified and observed for a total of 10.5 h (30 min observation period; *n* = 21) and 10 h (30 min per observation period; *n* = 20) in AGTE01 and AGTE02, respectively. More specifically, after a trophic egg was laid, we observed the fate of a given trophic egg as expressed by which caste a given trophic egg was offered to. As queens generally consumed trophic eggs much faster than other castes (Ito et al., unpublished data), we conducted a separate observation in which number of trophic eggs consumed by a given queen was recorded. Duration of observation was 10.5 h for each queen (30 min per observation period; *n* = 21).

### Statistical analysis

The gaster size and reproductive parameters (i.e., number of ovarioles/individual, number of yolky oocytes per ovarioles, and total number of yolky oocytes) between physogastric and normal workers were compared and analysed with Mann-Whitney *U*-test using SPSS version 16.0 (SPSS, Chicago, IL, USA) at 95% confidence interval. The same test was also applied to examine the morphometric differences between worker- and queen-produced offspring.

## Additional files

Additional file 1: Figure S1. Map of Taiwan showing the collection sites of three queenright colonies (AGQR01–03) used in the current study. (TIFF 63 kb)

Additional file 4: Figure S2. Physogastric workers in royal chamber. Physogastric workers were found tending younger brood (a) and form a dense retinue around the queen (b). (JPEG 14314 kb)
