# Supplementary figures and images for: Worker reproduction of the invasive yellow crazy ant Anoplolepis gracilipes
Source: Front Zool. 2017 May 8;14:24. doi: 10.1186/s12983-017-0210-4 (PMC5422973; doi:10.1186/s12983-017-0210-4)

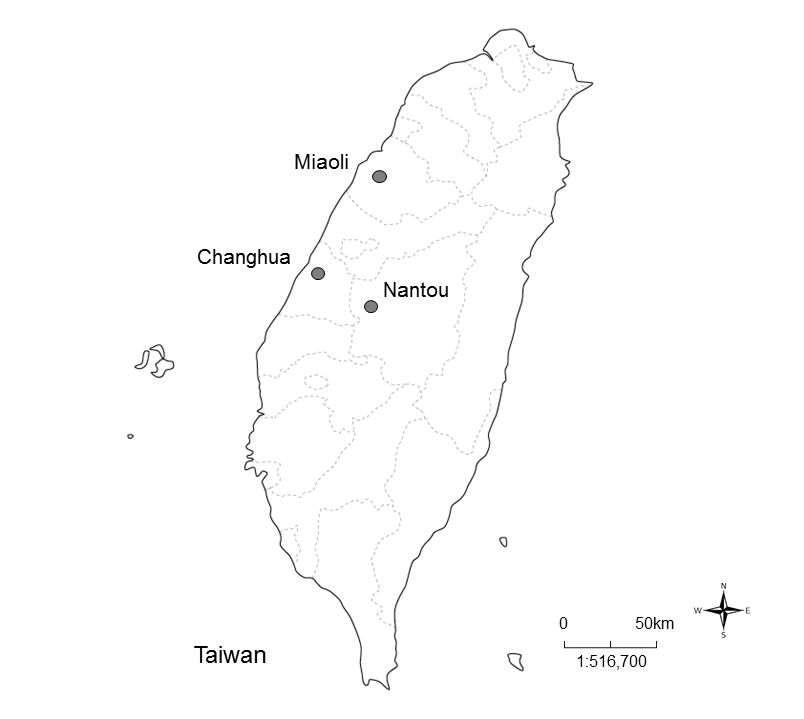

Supplement: Supplementary file 3 — Map of Taiwan showing the collection sites of three queenright colonies (AGQR01–03) used in the current study. (TIFF 63 kb) [file 12983_2017_210_MOESM1_ESM.tif]

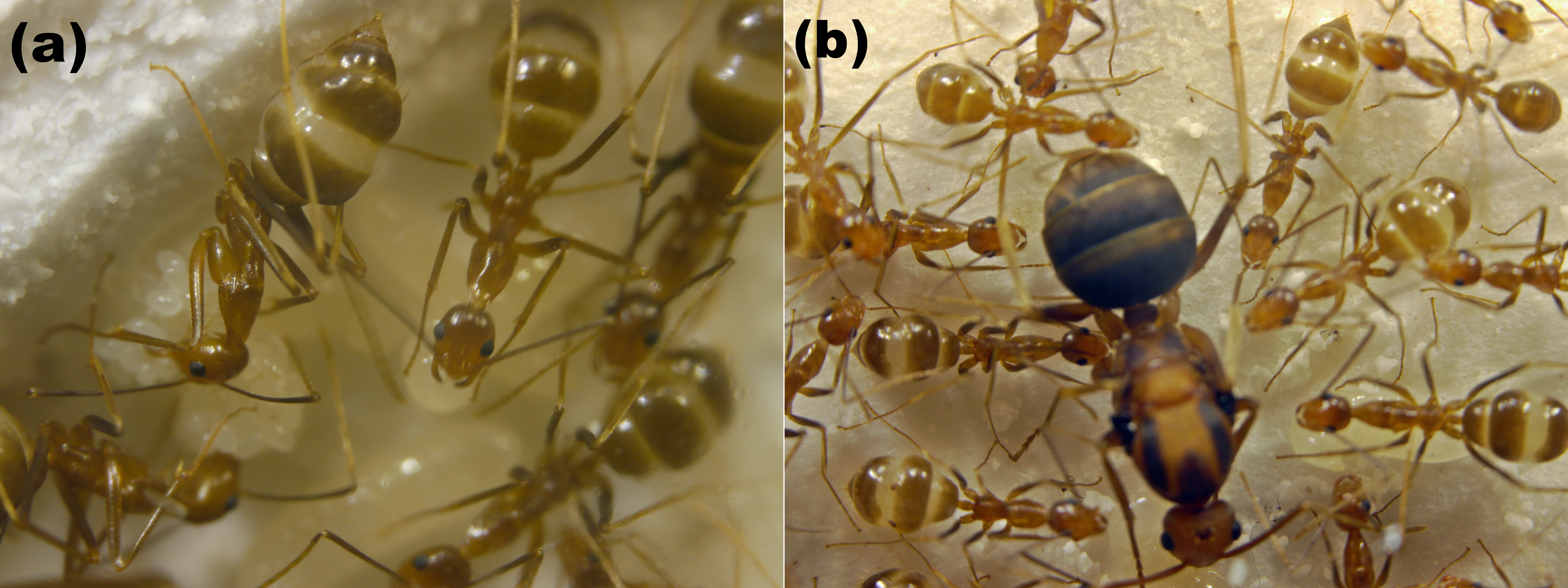

Supplement: Supplementary file 4 — Physogastric workers in royal chamber. Physogastric workers were found tending younger brood (a) and form a dense retinue around the queen (b). (JPEG 14314 kb) [file 12983_2017_210_MOESM4_ESM.jpg]
